# Proteomic Profiling of Plasma Biomarkers Associated With Return to Sport Following Concussion: Findings From the NCAA and Department of Defense CARE Consortium

**DOI:** 10.3389/fneur.2022.901238

**Published:** 2022-07-19

**Authors:** Rany Vorn, Sara Mithani, Christina Devoto, Timothy B. Meier, Chen Lai, Sijung Yun, Steven P. Broglio, Thomas W. McAllister, Christopher C. Giza, Hyung-Suk Kim, Daniel Huber, Jaroslaw Harezlak, Kenneth L. Cameron, Gerald McGinty, Jonathan Jackson, Kevin M. Guskiewicz, Jason P. Mihalik, Alison Brooks, Stefan Duma, Steven Rowson, Lindsay D. Nelson, Paul Pasquina, Michael A. McCrea, Jessica M. Gill

**Affiliations:** ^1^School of Nursing, Johns Hopkins University, Baltimore, MD, United States; ^2^National Institute of Nursing Research, National Institutes of Health, Bethesda, MD, United States; ^3^School of Nursing, University of Texas Health Science Center San Antonio, San Antonio, TX, United States; ^4^Henry M. Jackson Foundation for the Advancement of Military Medicine, Bethesda, MD, United States; ^5^Department of Neurosurgery, Medical College of Wisconsin, Milwaukee, WI, United States; ^6^Center for Neuroscience and Regenerative Medicine, Uniformed Services University of the Health Sciences, Bethesda, MD, United States; ^7^Predictiv Care, Mountain View, CA, United States; ^8^Michigan Concussion Center, University of Michigan, Ann Arbor, MI, United States; ^9^Department of Psychiatry, Indiana University School of Medicine, Indianapolis, IN, United States; ^10^Departments of Pediatrics and Neurosurgery, University of California, Los Angeles, Los Angeles, CA, United States; ^11^UCLA Steve Tisch BrainSPORT Program, University of California, Los Angeles, Los Angeles, CA, United States; ^12^Department of Epidemiology and Biostatistics School of Public Health-Bloomington, Indiana University, Bloomington, IN, United States; ^13^John A. Feagin Sports Medicine Fellowship, Keller Army Hospital, West Point, NY, United States; ^14^United States Air Force Academy, Colorado Springs, CO, United States; ^15^Matthew Gfeller Center, Department of Exercise and Sport Science, University of North Carolina at Chapel Hill, Chapel Hill, NC, United States; ^16^Department of Orthopedics, Division of Sports Medicine, School of Medicine and Public Health, University of Wisconsin-Madison, Madison, WI, United States; ^17^Department of Biomedical Engineering and Mechanics, Virginia Tech, Blacksburg, VA, United States; ^18^Department of Neurology, Johns Hopkins University, Baltimore, MD, United States

**Keywords:** sport injuries, concussion, return to sport (RTS), biomarker, proteomic

## Abstract

**Objective:**

To investigate the plasma proteomic profiling in identifying biomarkers related to return to sport (RTS) following a sport-related concussion (SRC).

**Methods:**

This multicenter, prospective, case-control study was part of a larger cohort study conducted by the NCAA-DoD Concussion Assessment, Research, and Education (CARE) Consortium, athletes (*n* = 140) with blood collected within 48 h of injury and reported day to asymptomatic were included in this study, divided into two groups: (1) recovery <14-days (*n* = 99) and (2) recovery ≥14-days (*n* = 41). We applied a highly multiplexed proteomic technique that uses DNA aptamers assay to target 1,305 proteins in plasma samples from concussed athletes with <14-days and ≥14-days.

**Results:**

We identified 87 plasma proteins significantly dysregulated (32 upregulated and 55 downregulated) in concussed athletes with recovery ≥14-days relative to recovery <14-days groups. The significantly dysregulated proteins were uploaded to Ingenuity Pathway Analysis (IPA) software for analysis. Pathway analysis showed that significantly dysregulated proteins were associated with STAT3 pathway, regulation of the epithelial mesenchymal transition by growth factors pathway, and acute phase response signaling.

**Conclusion:**

Our data showed the feasibility of large-scale plasma proteomic profiling in concussed athletes with a <14-days and ≥ 14-days recovery. These findings provide a possible understanding of the pathophysiological mechanism in neurobiological recovery. Further study is required to determine whether these proteins can aid clinicians in RTS decisions.

## Introduction

Each year, an estimated 8 million collegiate athletes participate in organized sports in the United States (1). The increasing sports-related concussion (SRC) rate has become a major public health concern due to the subtle nature of the injury, which makes diagnosis and return to sport (RTS) decision-making challenging (2). Premature RTS may adversely affect the neurobiological recovery process and increase risk for subsequent SRC, which has been linked to short or long-term neurological consequences (3, 4). Repetitive SRC has been associated with memory and cognitive deficits and risks of chronic traumatic encephalopathy (5, 6). The lack of prognostic biomarkers of recovery poses a significant challenge concerning assessing an athlete's readiness for RTS. Biomarkers are needed to guide clinical decision-making, thereby mitigating risk for exposure to head impacts before resolution of prior SRC. Concerning individuals at risk for prolonged recovery and poor outcomes, prognostic biomarkers as well as objective measures of neurobiological recovery are critically needed to facilitate early risk-stratification and ensure effective clinical management of SRC.

Peripheral blood-based biomarkers can play a vital role in identifying the intrinsic factors influencing SRC symptoms and recovery time. Serum glial fibrillary acidic protein (GFAP) and ubiquitin c-terminal hydrolase L1 (UCH-L1) have been shown to be elevated after a concussion, with higher levels observed in athletes with more severe acute injury characteristics (e.g., loss of consciousness, posttraumatic amnesia) (7). Further, both GFAP and tau have been associated with prolonged recovery (i.e., ≥14-days) after SRC (8). In addition to brain-specific biomarkers, altered inflammatory cytokine levels at acute and subacute timepoints can serve as prognostic markers of the neurobiological recovery. A recent study showed that higher levels of serum cytokines interleukin (IL) 1 receptor antagonist, and IL-6 within 6 h of injury were associated with greater symptom duration in concussed athletes (9). At the subacute timepoint, plasma monocyte chemoattractant protein-1 and 4 proteins were associated with recovery in SRC (10). Although these studies provide evidence that protein changes relate to SRC occurrence and recovery time, they are limited by a hypothesis-based approach that limits the identification of unique biomarkers changes that might provide a more in-depth characterization of relationships.

To date, biomarker studies related to RTS decisions have relied upon a hypothesis driven-approach, screening for markers with known involvement with traumatic brain injury (TBI) pathophysiology. Therefore, we implemented a proteomic approach to discover the candidate biomarkers for neurobiological recovery at 14 days or more after SRC occurrence. In addition, our study was to examine the protein networks and potential pathways involving neurobiological recovery. A 14-day cutoff was based on NCAA guidelines for management of SRC (11). We used the SOMAscan assay from SomaLogic to measure more than a thousand proteins in a scalable manner in a single specimen, which greatly accelerates biomarker discovery (12).

## Materials and Methods

### Participants and Clinical Data

A total of 140 concussed athletes include in this study were part of a larger cohort enrolled in the NCAA–Department of Defense Concussion Assessment, Research and Education (CARE) Consortium. The CARE Consortium was approved by the Medical College of Wisconsin Institutional Review Board and the Human Research Protection Office at the US Army Medical Research and Material Command and written informed consent was obtained for all participants. Concussion was defined according to the consensus definition from the US Department of Defense evidence based-guidelines (13). After an athlete was diagnosed with concussion, a single blood plasma sample was collected within 48 h, and a clinical battery consisting of the Sport Concussion Assessment Tool-Third Edition (SCAT-3), the Standardized Assessment of Concussion (SAC), the Balance Error Scoring System (BESS), and the Brief Symptom Inventory 18 (BSI-18). Concussed athletes were divided into two groups on the basis of the number of days following SRC that asymptomatic status was reported: (1) recovery <14-days, *n* = 99 and (2) recovery ≥14-days, *n* = 44. The group were matched by age, sex, height, weight, race, ethnicity, and years of participation.

### Blood Sampling Processing

A single timepoint of non-fasting blood samples were collected by venipuncture from athletes within 48 h following injury. Blood was collected in 10 ml plastic dipotassium ethylenediaminetetraacetic acid (EDTA) tubes, immediately placed on ice, centrifuged (15 min, 1,500 g, room temperature), and frozen (−27°C) in aliquots within 60 min of sample collection. Samples were shipped on dry ice to CARE Consortium biorepository at Indiana University School of Medicine and were stored at −80°C until analysis.

### SOMAscan Assay

Proteomic analysis was performed in plasma samples using the SOMAscan assay (SomaLogic Inc.), an aptamer-based assay that allows for the simultaneous measurement and quantification of a large number of proteins (1,305 proteins based on the version used here). The assay uses chemically modified nucleotides to transform a protein signal into a nucleotide signal quantified using relative fluorescence on microarrays (14, 15). Sample identifications were randomized in the SOMAscan plate prior to loading to minimize the potential for artifacts due to specific loci in a microarray and following the recommended sample preparation protocols. Samples were adequately stored in −80°C freezer before use, thawed, and then loaded into the barcoded vials within the plate using pipette techniques and 55 μl aliquots, keeping all samples and the plate on ice (4°C) throughout the process. The plate was analyzed by SomaLogic, Inc. and the standardization results were produced as relative fluorescent units (RFUs). Standardized results consisted of the following steps: hybridization control normalization, median signal normalization, and calibration normalization. Sample data were first normalized to remove hybridization variation within a run. Median signal normalization was performed across calibrator samples to remove other assay biases within the run. Overall scaling was then performed on a per-plate basis to remove overall intensity differences between runs. Calibration was then performed to remove assay differences between runs.

### Statistical Analysis

Data were analyzed using SPSS statistical software (Armonk, NY: IBM Corp.) and GraphPad prism 8.4 was used to generate graphs (La Jolla, CA: GraphPad Software). Descriptive statistics were used to describe the demographic and clinical characteristics of the participants. Chi-square (χ^2^) test and Mann–Whitney *U*-test were performed to determine the group differences. The log-fold changes were calculated by using an interactive Shiny web tool framework developed by the National Institutes of Health's Center for Human Immunology, Autoimmunity, and Inflammation (CHI) facility (16). Adjusted *p*-values were calculated by using Benjamini–Hochberg's false discovery rate (FDR). Dysregulated proteins were uploaded into the Ingenuity Pathway Analysis (IPA) software (Qiagen IPA) to explore the mechanistic networks most significantly associated with the study outcome. The significance level was set at 0.05 in all tests.

## Results

The demographic and clinical characteristics for concussed athletes with recovery <14-days and ≥14-days are shown in Table 1. The recovery group ≥14-days and <14-days group were between 17 and 23 years of age. The groups did not significantly differ on age, sex, race, and ethnicity. The median number of days to attainment of asymptomatic status for the <14-days group was 4.9 days and 18.9 days for ≥14-days group. Concussed athletes with recovery ≥14-days had significantly higher SCAT symptom severity scores, BESS total scores, and BSI-18 global severity scores compared with the recovery <14-days group.

**Table 1:** Sample characteristics of RTS athletes.

	** <14-days (*n* = 99)**	**≥14-days (*n* = 41)**	***P***
Demographics and history			
Age, mean (SD), y	18.7 (1.1)	18.7 (1.2)	0.867
Male, *N* (%)	82 (82.8)	29 (70.7)	0.108
Height, mean (SD), total in.	71.4 (3.9)	70.6 (4.4)	0.341
Weight, mean (SD), lbs	193.3 (45.7)	185.8 (47.1)	0.381
Years of sport participation, mean (SD), y	10.5 (3.9)	9.8 (4.4)	0.455
Race, *N* (%)			0.072
White	67 (68.4)	28 (70.0)	
Black	22 (22.4)	3 (7.5)	
Asian	3 (3.1)	5 (12.5)	
Hawaiian or Pacific Islander	1 (1.0)	1 (2.5)	
Multiple	5 (5.1)	3 (7.5)	
Ethnicity, *N* (%)			0.639
Non-hispanic	82 (82.8)	33 (82.9)	
Hispanic	4 (4.0)	3 (7.3)	
Unknown/not reported	13 (13.1)	4 (9.8)	
Days to asymptomatic, mean (SD), d	6.3 (4.1)	22.2 (5.5)	<0.001
Median (min.-max.)	4.9 (0.8–13.1)	18.9 (14.0–87.6)	
Median (IQR)	4.9 (7.8)	18.9 (7.6)	
Time to blood draw (hour), mean (SD)	18.4 (14.1)	16.9 (15.2)	0.574
SCAT symptom severity score, mean (SD)	25.2 (17.2)	37.4 (19.2)	<0.001
SAC total score, mean (SD)	26.7 (2.5)	26.7 (1.7)	0.975
BESS total error, mean (SD)	14.3 (7.1)	18.3 (8.8)	0.016
BSI global severity score, mean (SD)	3.7 (4.4)	7.4 (6.3)	0.002

We found 87 proteins that were significantly dysregulated in recovery ≥14-days relative to recovery <14-days. The top proteins upregulated in recovery ≥14-days were haptoglobin (HP), leptin (LEP), apolipoprotein B-100 (APOB), tyrosine kinase 2 (TYK2), advanced glycosylation end product-specific receptor (AGER), and IL36A. Top proteins that were downregulated in recovery ≥14-days were erythrocyte membrane protein band 4.1 (EPB41), protein S100-A12 (S100A12), WNK lysine deficient protein kinase 3 (WNK3), ATP synthase subunit beta, mitochondrial (ATP5B), and epidermal growth factor (EGF) (Table 2). All proteins that were significantly different in concussed athletes with recovery <14-days relative to recovery ≥14-days are listed in the Supplementary Table 1.

**Table 2:** Protein expression differences between recovery ≥14-days and <14-days.

**Target**	**Target protein name**	**Log-fold change**	***P*-value**
**Upregulated**			
HP	Haptoglobin	0.630	0.019
LEP	Leptin	0.541	0.004
APOB	Apolipoprotein B-100	0.390	0.028
TYK2	Tyrosine kinase 2	0.386	0.040
AGER	Advanced glycosylation end product-specific receptor	0.291	0.027
IL36A	Interleukin 36 alpha	0.271	0.047
FGF23	Fibroblast growth factor 23	0.270	0.027
CD5L	CD5 antigen-like	0.265	0.015
LMAN2	Lectin, mannose binding 2	0.261	0.030
CTSF	Cathepsin F	0.217	0.004
IL12B.IL23A	Interleukin-23	0.203	0.030
ENTPD5	Ectonucleoside triphosphate diphosphohydrolase 5	0.197	0.016
ICAM5	Intercellular adhesion molecule 5	0.196	0.021
IDUA	Alpha-L-iduronidase	0.193	0.045
GPC3	Glypican 3	0.186	0.028
**Downregulated**			
EPB41	Erythrocyte membrane protein band 4.1	−0.482	0.043
S100A12	Protein S100-A12	−0.449	0.030
PGD	Phosphogluconate dehydrogenase	−0.411	0.028
WNK3	WNK lysine deficient protein kinase 3	−0.405	0.007
ATP5B	ATP synthase subunit beta, mitochondrial	−0.324	0.047
EGF	Epidermal growth factor	−0.320	0.002
IMPDH1	Inosine-5'-monophosphate dehydrogenase 1	−0.318	0.036
VWF	Von Willebrand factor	−0.317	0.008
PPA1	Inorganic pyrophosphatase	−0.292	0.040
RPS6KA3	Ribosomal protein S6 kinase A3	−0.281	0.032
FGF6	Fibroblast growth factor 6	−0.276	0.010
CA3	Carbonic anhydrase 3	−0.275	0.023
FGF8	Fibroblast growth factor 8	−0.249	0.031
FN1	Fibronectin 1	−0.248	0.042
IFNL1	Interferon lambda-1	−0.244	0.003

**Figure 1:**
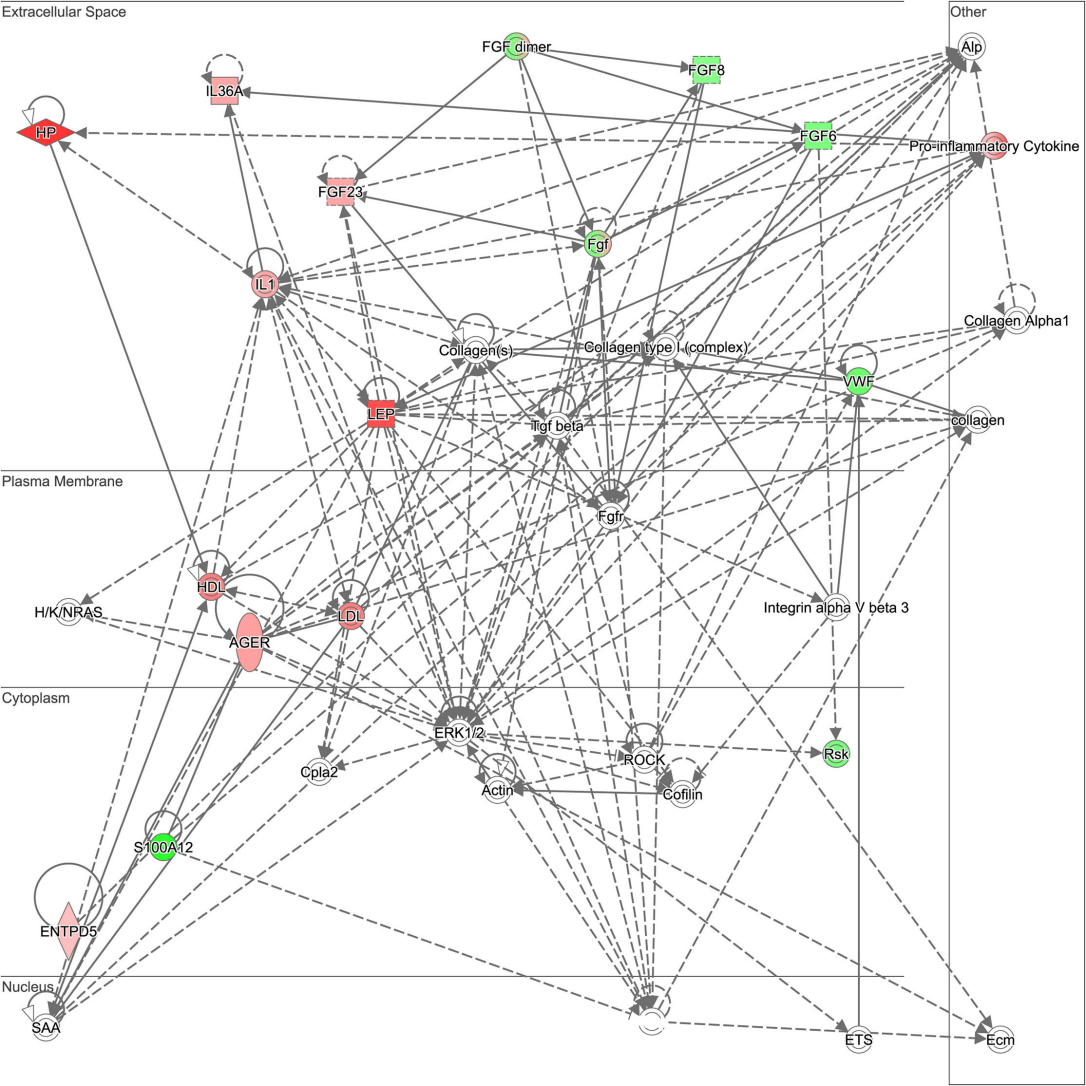
Ingenuity Pathway Analysis (IPA) mechanistic network. Top Ingenuity Pathway Analysis (IPA) mechanistic network analysis revealed the protein-protein interaction in the network associated with Hepatic System Development and Function, Cellular Movement, and Organismal Injury and Abnormality. Green indicates that the protein is downregulated and red indicates that the protein is upregulated, with increased color saturation representing more extreme measurement in the dataset. Proteins in gray indicate the direct relationship with differential expression proteins in the network. Solid lines indicate direct interaction and dashed lines indicated indirect interactions.

The 30 proteins that were most significantly dysregulated were uploaded into IPA software for core expression analysis. The pathway analysis revealed the molecular mechanisms underlying the prolonged recovery in SRC. The top enrichment of the canonical pathway and the associated proteins in each pathway are presented in Table 3. Top canonical pathways associated with recovery were Signal Transducer and Activator of Transcription 3 (STAT3) Pathway, Tumor Microenvironment Pathway, Regulation of the Epithelial Mesenchymal Transition by Growth Factors Pathway, and Acute Phase Response Signaling. Top IPA mechanistic network analysis revealed the protein-protein interaction in the network associated with Hepatic System Development and Function, Cellular Movement, and Organismal Injury and Abnormalities (Figure 1).

**Table 3:** Top canonical pathway associated with recovery.

**Ingenuity canonical pathways**	**–log(*p*-value)**	**Ratio**	**Molecules**
STAT3 pathway	9.72	0.0667	EGF,FGFR1,IL12RB2,IL15RA,IL18R1,IL6R,KRAS,PDGFRA,TYK2
Tumor microenvironment pathway	8.63	0.0503	ARG1,EGF,FGF23,FGF6,FN1,IL6R,KRAS,LEP,PROK1
Regulation of the epithelial mesenchymal transition by growth factors pathway	7.08	0.0417	EGF,FGF23,FGF6,FGFR1,IL6R,KRAS,PDGFRA,TYK2
Acute phase response signaling	5.97	0.0378	FN1,HP,IL36A,IL6R,KRAS,SERPIND1,VWF
Th1 pathway	5.84	0.0492	DLL4,IL12RB2,IL18R1,IL6R,NOTCH3,TYK2
Hepatic fibrosis/hepatic stellate cell activation	5.83	0.0361	EGF,FGFR1,FN1,IL6R,LEP,PDGFRA,PROK1
Regulation of the epithelial-mesenchymal transition pathway	5.82	0.0359	EGF,FGF23,FGF6,FGFR1,KRAS,NOTCH3,TYK2
PI3K/AKT signaling	5.76	0.0352	CDKN1B,IL12RB2,IL15RA,IL18R1,IL6R,KRAS,TYK2
Cardiac hypertrophy signaling (enhanced)	5.5	0.0186	FGF23,FGF6,FGFR1,IL12RB2,IL15RA,IL18R1,IL36A,IL6R,KRAS,LEP
Glucocorticoid receptor signaling	5.2	0.0172	ATP5F1B,EGF,HP,IL12RB2,IL15RA,IL18R1,IL6R,KRAS,PLA2G2A,TYK2
Actin cytoskeleton signaling	5.16	0.0286	EGF,FGF23,FGF6,FN1,GSN,KRAS,PAK5
Th1 and Th2 activation pathway	4.98	0.0349	DLL4,IL12RB2,IL18R1,IL6R,NOTCH3,TYK2
Bladder cancer signaling	4.66	0.0431	EGF,FGF23,FGF6,KRAS,PROK1
Pancreatic adenocarcinoma signaling	4.49	0.0397	CDKN1B,EGF,KRAS,PROK1,TYK2
ErbB2-ErbB3 signaling	4.42	0.0615	CDKN1B,ERBB3,KRAS,TYK2

## Discussion

The RTS decision is important for the safety of collegiate athletes to avoid the short- and long-term impact of repetitive concussions. Here, using a proteomic approach, we were able to identify several dysregulated plasma protein biomarkers in concussed athletes with recovery ≥14-days relative to concussed athletes with recovery <14-days. When evaluated within 48 h of injury, the SCAT-3, BESS, and BSI-18 scores were significantly higher in ≥14-days compared with <14-days. These findings provide a better understanding of pathophysiological mechanisms of sports injuries for exploring potential prognostic and recovery markers related to SRC.

Beyond the clinical implications of the findings, they also advance understanding of the pathophysiological mechanism in neurobiological recovery process following concussion. Pathway analysis showed that the most significant proteins were associated with STAT3 pathway. Signal transducer and activator of transcription (STAT3) is a member of the STAT family and deregulated STAT3 pathway may contribute to the proinflammatory response in SRC (17, 18). The proteins represented in this pathway are listed in Table 3. In addition, the proteins involved in the mechanistic network include LEP, HP, VWF, AGER, S100A4, fibroblast growth factor 6 (FGF6), and FGF23. In particular, LEP is a 16 kDa protein secreted predominantly by white adipose tissue which regulates energy homeostasis, neuroendocrine function, metabolism, and inflammatory response (19). In the context of brain injury, LEP and its receptor protein may be expressed from many brain regions, which may be involved in regulation of the central nervous system (CNS) (20). However, another plausible explanation is that LEP is released from adipocytes which may cross the blood brain barrier (BBB) to regulate many functions in the CNS such as synaptic plasticity and spatial learning and memory (21). The elevation of LEP levels in the prolonged recovery group is consistent with the findings of previous studies related to neurodegeneration. An observational study has identified an association between LEP level with cognitive decline in healthy older age population (22). Furthermore, cerebrospinal fluid LEP was higher in AD population compared with healthy controls (23). Conversely, not all studies support these findings, in a 15 years longitudinal study showed that higher levels of LEP proteins are associated with reduced incidence of dementia and Alzheimer's disease (AD) (24). Also, higher serum LEP levels at baseline are protective against cognitive decline after 5 years of follow-up in the healthy older population (25). As such, LEP protein may act as a neuroprotective role in the CNS system. In support of this, *in vitro* study suggested that LEP reduced tau phosphorylation levels (26), which is a hallmark of neurodegenerative diseases including AD and chronic traumatic encephalopathy through the aggregation of neurofibrillary tangles (27–29). A preclinical study of mice showed that LEP has a positive effect in neuronal protection against ischemic brain injury through extracellular signal-related kinase (ERK) 1/2 signaling pathway (30). LEP may have a therapeutic effect following spinal cord injury by enhancing the expression of neuroprotective and anti-inflammatory genes (31). Further study is needed to investigate the effect of LEP protein as a prognostic marker for concussion.

Disruption of the BBB following SRC may lead to increased release of proteins into peripheral circulation and recruitment of immune cells or proteins to the injury site (32). We identified several vascular injury markers such as vascular endothelial growth factor C (VEGFC), VWF, Platelet derived growth factor receptor alpha (PDGFRA), and FN1 proteins that may be involved in BBB damaged following concussion. In particular, VEGF plays an important role in angiogenesis and neuroprotection (33, 34), and is mainly expressed from endothelial cells to preserves intact BBB. Increased peripheral circulating levels of VEGF have been linked to alterations in BBB permeability following a TBI and infiltration of immune cells (35, 36). Our lab has recently demonstrated that VEGF protein was dysregulated in concussed athletes and associated with neuroimage findings in mild TBI patients (37). VWF is an acute phase protein response to vascular injury and has been reported in several diseases such as acute coronary syndrome (38), cancer (39), and TBI (40). Elevated plasma VWF was correlated with unfavorable outcomes in severe TBI patients (41). However, we found inconsistent results of plasma VWF proteins to previous studies. We reported lower VWF protein levels in the recovery ≥14-days group compared with recovery <14-days group. The difference in these findings may be attributed to different demographic population of the study, injury type (i.e., sports injury) and the type of protein assay used to quantify the data. Increased plasma FN1 proteins predominantly reflect vascular injury after a concussion, which has an important role in the repair of tissue damage and protection against neuron cell death by focal ischemia (42). A preclinical study has shown that TBI model with FN deficiency group had the worst motor and cognitive ability (43). Our data indicate that dysregulation of these protein expressions in concussed athletes with recovery ≥14-days may affect neuronal damage and delay neurobiological recovery after concussion.

Moreover, AGER is a multiligand receptor and a member of the immunoglobin superfamily which plays a major role in inflammation response in binding of its ligands, including amyloid beta, damage-associated molecule patterns, S100 proteins, and high-mobility group box-1 protein (44, 45). Elevated AGER concentration in our data may reflect the severity of injury which caused the prolonged recovery. Another plausible explanation is that imbalanced production of pro-inflammatory and neuroprotective protein that may cause secondary injury and lead to a worsened pathophysiological response following sport concussion. In support of this, our data have shown that S100A4 protein was significantly downregulated in concussed athletes with recovery ≥14-days. The effect of S100A4 protein expression following TBI in protecting against neuron cell death has been shown in the preclinical model (46). Downregulation of S100A4 protein expression may prolong the neurological recovery process. Also, we reported a higher expression of FGF23 in recovery ≥14-days group. FGF23 is a circulating hormone that regulates phosphate homeostasis and vitamin D metabolism. Elevated serum FGF23 concentration is a potential prognostic marker of chronic kidney disease (47), and is associated with dementia and AD (48). Moreover, elevated plasma FGF23 concentration has been implicated as a potential risk factor for stroke and intracerebral hemorrhage (49). Upregulation of FGF23 may serve as a prognostic marker of prolonged recovery.

This study has several limitations. First, our results may not be applicable to other populations (e.g., non-athletes, younger or older athletes). Our study participants were well matched between the group, although they had a relatively small number of athletes who sustained SRC with the prolonged recovery group. Due to the expense of the SOMAscan analysis, blood from a single time point was analyzed. Future studies should conduct this prognostic analysis as part of a longitudinal study with a larger cohort. Injury mechanisms may vary widely across the injury cohort and could plausibly impact the brain neurophysiology recovery as a result. Finally, these proteins were not significant after multiple corrections and the results should be interpreted cautiously.

Although this study has several limitations, the large-scale proteomic analysis strengthens our results. Our data represent the first exploratory biomarkers derived from a large-scale proteomic analysis to determine the neurobiological recovery after concussion. In addition, our findings indicate the feasibility and utility of proteomic profiling in identifying the blood-based biomarkers for neurobiological recovery following concussion. Our data provide a better understanding of physiological mechanisms associated with concussion recovery. Further research is required to determine the eventual clinical utility of these candidate proteins.

## Data Availability Statement

The raw data supporting the conclusions of this article will be made available by the authors, without undue reservation.

## Ethics Statement

The studies involving human participants were reviewed and approved by Medical College of Wisconsin Institutional Review Board and the Human Research Protection Office at the US Army Medical Research. Written informed consent to participate in this study was provided by the participants' legal guardian/next of kin.

## Author Contributions

RV, MM, CG, KC, PP, SB, JH, KG, TMc, and JG: conceptualization. RV, JG, CL, and H-SK: methodology. RV: software and visualization. RV, JG, CL, H-SK, SY, and CD: formal analysis. RV, CL, JG, MM, SB, and CG: investigation. JG, MM, CG, and PP: resources. RV and CD: data curation. RV and JG: writing—original draft preparation. RV, SM, CD, TMe, CL, SY, SB, TMc, CG, H-SK, DH, JH, KC, GM, JJ, KG, JM, AB, SD, SR, LN, PP, MM, and JG: writing—review and editing. JG: supervision. JG, SB, TMc, CG, DH, KG, JM, LN, and MM: project administration. JG, MM, SB, TMc, JM, and PP: funding acquisition. All authors contributed to the article and approved the submitted version.

## Funding

This research was supported by the Grand Alliance Concussion Assessment, Research, and Education (CARE) Consortium and funded in parts by the National Collegiate Athletic Association (NCAA) and the Department of Defense (DOD). The U.S. Army Medical Research Acquisition Activity, 820 Chandler Street, Fort Detrick MD 21702-5014 is the awarding and administering acquisition office. This work was supported by the Office of the Assistant Secretary of Defense for Health Affairs, through the Combat Casualty Care Research Program, endorsed by the Department of Defense, under Award No. W81XWH1420151. Opinions, interpretations, conclusions and recommendations are those of the author and are not necessarily endorsed by the DOD.

## Conflict of Interest

SY was employed by Predictiv Care, Inc. The remaining authors declare that the research was conducted in the absence of any commercial or financial relationships that could be construed as a potential conflict of interest.

## Publisher's Note

All claims expressed in this article are solely those of the authors and do not necessarily represent those of their affiliated organizations, or those of the publisher, the editors and the reviewers. Any product that may be evaluated in this article, or claim that may be made by its manufacturer, is not guaranteed or endorsed by the publisher.
